# Turning a Tumor Microenvironment Pitfall into Opportunity: Discovery of Benzamidoxime as PD-L1 Ligand with pH-Dependent Potency

**DOI:** 10.3390/ijms24065535

**Published:** 2023-03-14

**Authors:** Elisa Bianconi, Alessandra Riccio, Luana Ruta, Carlo Bigiotti, Andrea Carotti, Sonia Moretti, Bruno Cerra, Antimo Gioiello, Simone Ferlin, Efisio Puxeddu, Antonio Macchiarulo

**Affiliations:** 1 Department of Pharmaceutical Sciences, University of Perugia, Via del liceo n.1, 06123 Perugia, Italy; 2 Department of Medicine and Surgery, University of Perugia, P.le L. Severi, 06132 Perugia, Italy; 3 Sterling S.p.A., Via della Carboneria n.30, 06073 Corciano, Italy

**Keywords:** fragment-based drug design, molecular docking, biophysics, virtual screening, thermophoresis

## Abstract

PD-1/PD-L1 protein complex is attracting a great deal of interest as a drug target for the design of immune therapies able to block its assembly. Although some biologic drugs have entered clinical use, their poor response rate in patients are demanding further efforts to design small molecule inhibitors of PD-1/PD-L1 complex with higher efficacy and optimal physicochemical properties. Dysregulation of pH in the tumor microenvironment is indeed one of the key mechanisms promoting drug resistance and lack of response in cancer therapy. Integrating computational and biophysical approaches, herein we report a screening campaign that has led to identifying VIS310 as a novel ligand of PD-L1, with physicochemical properties enabling a pH-dependent binding potency. Additional optimization efforts by analogue-based screening have been instrumental to disclosing VIS1201, which exhibits improved binding potency against PD-L1 and is able to inhibit PD-1/PD-L1 complex formation in a ligand binding displacement assay. While providing preliminary structure–activity relationships (SARs) of a novel class of PD-L1 ligands, our results lay the foundation for the discovery of immunoregulatory small molecules resilient to tumor microenvironmental conditions for escaping drug-resistance mechanisms.

## 1. Introduction

Recent years have witnessed the development of immune checkpoint inhibitors (ICIs) as groundbreaking therapies in immuno-oncology. In this framework, the programmed cell death protein-1 (PD-1) and its ligands, PD-L1 and PD-L2, are among the most well-validated and effective immunotherapeutic targets in cancer, autoimmune disorders, and infectious diseases [1,2].

PD-1 is mainly expressed on activated mature T cells and on double-negative (CD4^−^CD8^−^) T cells, activated natural killer T cells, B cells, monocytes, and immature Langerhans cells to a lower extent [3]. PD-L1 is constitutively expressed at low levels on antigen-presenting cells (APCs) and on non-hematopoietic cell types, such as vascular endothelial cells, pancreatic islet cells, and cells of immune privileged tissues (e.g., placenta, testes, and eye). PD-L1 is also expressed on several cancer cells [4]. 

The formation of PD-1/PD-L1 complex triggers a co-inhibitory stimulus that blocks downstream signaling from T-cell receptors (TCRs) and CD28-mediated responses [5]. As a result, T cells become exhausted producing fewer cytokines, ceasing to differentiate and proliferate, and losing their ability to perform cytolytic and effector functions. It has been shown that the secretion of IFNγ by tumor-infiltrating T cells results in the overexpression of PD-L1 in tumor cells, allowing cancer to escape from the immune system’s response [6]. The presence of PD-L1 in the tumor microenvironment is thus correlated with poor prognosis in multiple cancer types [7]. Hence, blocking the PD-1/PD-L1 interaction can reverse immunosuppressive conditions and boost the recognition of tumor cells for destruction by the immune system [8]. 

**Figure 1:**
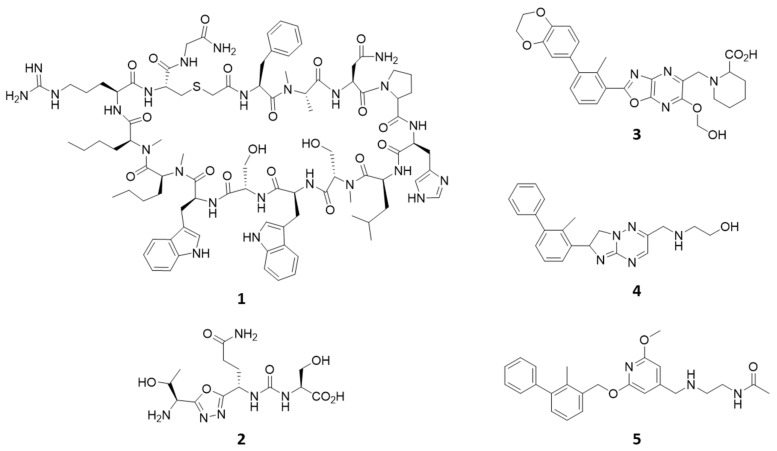
Examples of chemical structures of cyclic peptides (**1**), peptidomimetics (**2**), and biphenyl derivatives (**3**–**5**) reported as PD-1/PD-L1 inhibitors.

Over the last years, monoclonal antibodies (mAbs) against both PD-1 and PD-L1 have been developed and approved by the FDA as biologic drugs for cancer treatment [9,10]. However, despite representing breakthrough therapies in immuno-oncology, they suffer from limitations, in part due to their high molecular weight [11,12]. mAbs have high costs of production and limited bioavailability. They are also potentially immunogenic because of their long half-life, which may result in immune-related adverse events (iRAEs). Even more, they often exhibit a poor and variable response rate among cancer patients, likely due to the high incidence of drug resistance mechanisms [13,14]. Although some efforts have been devoted to the design of small molecule inhibitors of the PD-1/PD-L1 complex with the aim of overcoming mAbs limitations [15,16,17], their development as a novel anticancer immunotherapy is still lagging. Chemical classes of PD-1/PD-L1 inhibitors include macrocyclic peptides, peptidomimetics, and compounds bearing a biphenyl moiety that bind to PD-L1 (Figure 1) [18,19,20,21,22,23]. 

**Figure 2:**
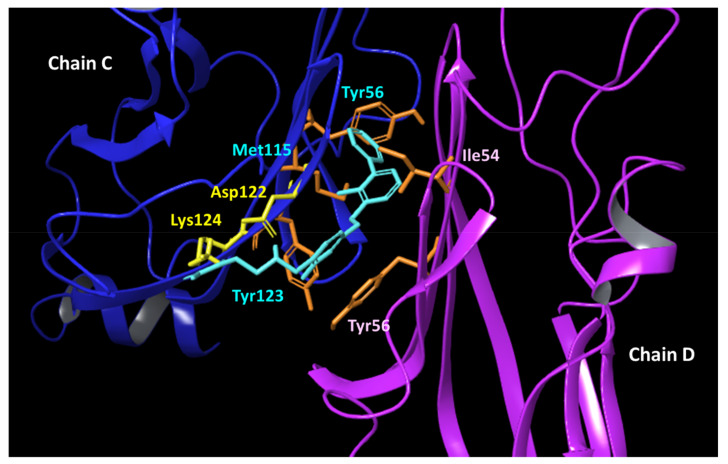
Crystal structure of PD-L1 dimer (chains C and D) bound to BMS-202 (**5**; PDB code 5J89). Ligand (**5**) is shown with cyan sticks. Hydrophobic residues of the binding cavity are highlighted in orange sticks (chain C: Tyr56, Met115, Tyr123; chain D: Ile54, Tyr56). Residues Asp122 and Lys124 (chain C) are shown with yellow sticks.

Crystallographic studies have unveiled that such ligands bind to a shallow surface of PD-L1, composed of GFC strands, by establishing hydrophobic interactions [24]. In particular, biphenyl compounds (e.g., BMS-202, **5**; Figure 2) induce a dimeric form of the target protein, in which they occupy a hydrophobic cavity shaped by two GFC surfaces of distinct PD-L1 units [18].

The wide hydrophobic and shallow surface of the binding site is a challenging aspect for designing drug-like inhibitors of the PD-1/PD-L1 interaction for clinical development. More recently, we have shown that pH shifts, as those occurring in the tumor microenvironment upon lactic acid production [25,26], represent a further obstacle, affecting the binding properties of PD-L1 ligands [27]. 

The dysregulation of pH in the tumor microenvironment is an important factor promoting cancer progression, drug resistance, and a lack of response in cancer therapy [28,29], including pancreatic cancer [30]. 

While supporting the alteration of pH as a part of the complex drug resistance mechanisms of tumor cells, our study also provided some insights into the optimal structural features and suitable physicochemical properties for designing drug-like PD-L1 ligands as inhibitors of the PD-1/PD-L1 complex that may be resilient to tumor microenvironmental pH conditions. In particular, the relevance of functional groups in ligands that are able to establish electrostatically enforced hydrogen bonds with the residues Asp122 and Lys124 of PD-L1 was observed. This observation adds to the previous findings of crystallographic studies that indicate hydrophobic contacts and aromatic interactions as pivotal to promoting ligand binding to PD-L1 [24,31].

Therefore, designing small molecule inhibitors of the PD-1/PD-L1 complex with physicochemical properties enabling pH-dependent binding activity could be a promising strategy to improve the efficacy, as well as the specificity, of immunotherapy against types of cancer endowed with dysregulated pH conditions in the tumor microenvironment.

As a follow-up to our efforts devoted to studying the molecular aspects of ligand recognition by PD-L1, in this work, we report a structure-based screening campaign of small molecules (molecular weight, MW ≤ 500 dalton) to identify novel hit compounds endowed with pH-dependent binding potency and the ability to disrupt the PD-L1/PD-1 interaction. Specifically, the campaign integrates molecular docking studies, exploiting the new hot-spot of interactions (Asp122 and Lys124) on the target protein, and microscale thermophoresis (MST) experiments designed to prove the binding activity of selected compounds to PD-L1, as well as the ability to inhibit the formation of the PD-1/PD-L1 complex.

## 2. Results

### 2.1. Selection of Virtual Hit Compounds

A chemical library containing a total of 5801 small molecules was assembled, enriching an internal subset of compounds (807 molecules, MW ≤ 500 dalton) with an external subset of highly soluble fragments from Life Chemicals (4994 molecules, MW ≤ 300 dalton). After refining the chemical structures, as detailed in the methods section, the chemical library was docked into the binding site close to residues Asp122 and Lys124 of the PD-L1 structure (PDB code 5J89) [24]. The binding energy was evaluated using GScore (kcal/mol), and virtual hit compounds were sorted according to better GScore (cutoff ≤ −4 kcal/mol) and prioritized for the presence of electrostatic and/or hydrogen bond interactions with Asp122 and/or Lys124 residues in the resulting binding mode. A first round of selection was accomplished by picking the top scored 50 compounds belonging to the internal subset of readily available small molecules. Then, a second round of selection was done by picking the top scored 10 compounds belonging to the subset of highly soluble fragments to be purchased. This second group of compounds was also endowed with a high structural diversity from the first group of compounds, as evidenced by a multidimensional scaling analysis performed with ChemMine using atom pair similarity measures and transforming the generated Tanimoto coefficients into distance values (Figure S1 and Table S1, supplementary material) [32]. Hence, a total of 60 compounds was selected for experimental binding assays with MicroScale Thermophoresis (MST).

### 2.2. Identification of VIS310 as Hit Compound

After confirming the stability of the protein in the experimental conditions of the study (Table S2, supplementary material), single-point binding assays against the fluorescently labeled PD-L1 (NT650/PD-L1) were carried out with MST. Specifically, each compound was tested at a concentration of 250 µM, along with BMS-202 (**5**) that was used as a positive control at the same concentration. Putative hit compounds were defined as those molecules inducing a thermophoretic movement of the ligand-bound complex with a fluorescent signal (F-norm) outside the signal value and three-fold standard deviations of the negative control (vehicle, DMSO). 

**Figure 3:**
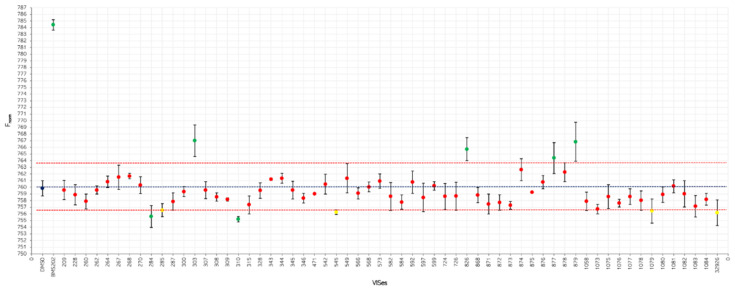
Results of single-point binding assays against NT650/PD-L1 of 60 virtual hits (Table S1, supplementary material) as compared to vehicle (DMSO) and positive control (BMS-202, **5**). Green and red dots indicate virtual hits compliant and non-compliant to the definition criterion of binders, respectively; yellow dots indicate compounds at the borderline of the definition criterion.

The inspection of the results (Figure 3) reveals 6 hit compounds (**6**, **8**, **9**, **11**–**13**) and 4 molecules laying at the borderline of the definition criterion (**7**, **10**, **14**, **15**). These compounds were next tested in full ligand binding assays against NT650/PD-L1 at pH 7.2, using the scalar concentration of each compound to obtain binding curves and the relative dissociation constants (K_d_). 

As a result, only one compound, namely, VIS310 (**9**), was able to generate a dissociation constant in the high-micromolar range of potency (K_d_ = 163.75 ± 33.61 μM; Table 1), though with a better binding efficiency index (BEI = 21.0) than BMS-202 (**5**) (K_d_ = 8.13 ± 1.38 μM; BEI = 12.1).

**Figure 4:**
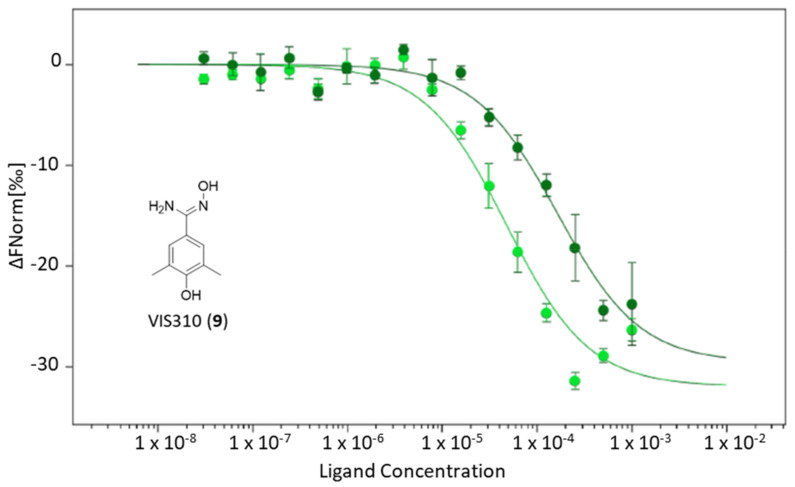
MST binding curves of VIS310 (**9**) to NT650/PD-L1 at pH 7.2 (dark green circles and line) and 6.2 (light green circles and line).

We previously reported that the binding activity of BMS-202 (**5**) is affected by the pH condition of the experiment, with lower pH values strengthening its interaction with PD-L1 (pH 6.2; K_d_ = 4.50 ± 0.56 μM; BEI = 12.8) [27]. Accordingly, a second binding experiment of VIS310 was carried out against PD-L1 at pH 6.2, resulting in a significant improvement of the ligand binding activity (pH 6.2, K_d_ = 47.81 ± 11.50 μM; BEI = 24.0; Figure 4). 

The chemical structure of VIS310 (**9**) is based on a substituted benzamidoxime scaffold. Accordingly, an analogue-based search was conducted to identify additional compounds containing this scaffold as core structure for experimental binding assays with MST. 

### 2.3. VIS310 Analogues and Structure–Activity Relationships

**Figure 5:**
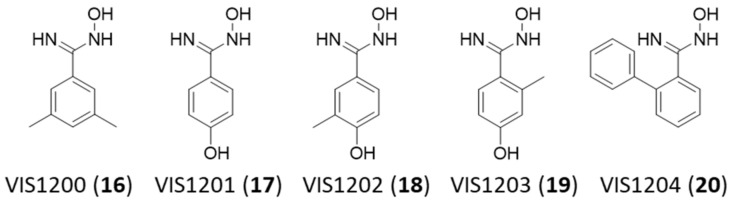
Chemical structures of VIS310 analogues (**16**–**20**).

The benzamidoxime scaffold was used as the query substructure to identify VIS310 analogues amid compounds of the chemical library (5801 molecules) used in the structure-based screening campaign. Since no result was obtained, a second search was performed using a larger chemical library, namely, the REAL database of Enamine, containing about 43.8 million drug-like compounds. As a result, five analogues of VIS310 (**9**) were found bearing different hydroxyl, methyl, and phenyl groups on the benzene moiety of the benzamidoxime scaffold (compounds **16**–**20**, Figure 5). These compounds were tested in full ligand binding assays against NT650/PD-L1 at both the conditions of pH 7.2 and 6.2. Results are reported in Table 2. 

The inspection of the dissociation constants and BEI values shows again a consistent improvement of the ligand binding activity when compounds are tested at pH 6.2, supporting a pH-dependent potency. Of note, different structure–activity relationship (SAR) schemes can be drawn at the two pH values. At pH 6.2, the removal of the *para* hydroxyl group in VIS1200 (**16)** determines a significant drop of the binding activity, suggesting the relevance of such polar group for the binding potency of the ligand to PD-L1. Conversely, the removal of one methyl group or the shift of its position from *meta* to *ortho* marginally affects the activity. Removal of both methyl groups keeps a similar binding activity to the parent compound, thus improving the BEI of the analogue. Finally, the presence of a phenyl moiety at the *ortho* position of the benzamidoxime scaffold is detrimental for the activity. 

In contrast, at pH 7.2, most of the analogues show a significant deterioration of the binding potency when compared to VIS310 (**9**), except for VIS1204 (**20**), wherein the presence of the *ortho* phenyl substituent allows a marginal improvement of the binding activity to PD-L1. Hence, the higher pH value in the ligand binding experiments seems to weaken the binding interactions of most of the analogues to the target protein. In the case of the more lipophilic VIS1204 (**20**), it is likely that hydrophobic interactions prevail over polar interactions at pH 7.2, accounting for the slight improvement of the binding activity of this analogue. To gain more insights into such pH-dependent binding potency, knowledge of the acidic dissociation constants (pKa) of VIS310 (**9**) analogues is of utmost importance. 

### 2.4. pKa Measurements

**Figure 6:**
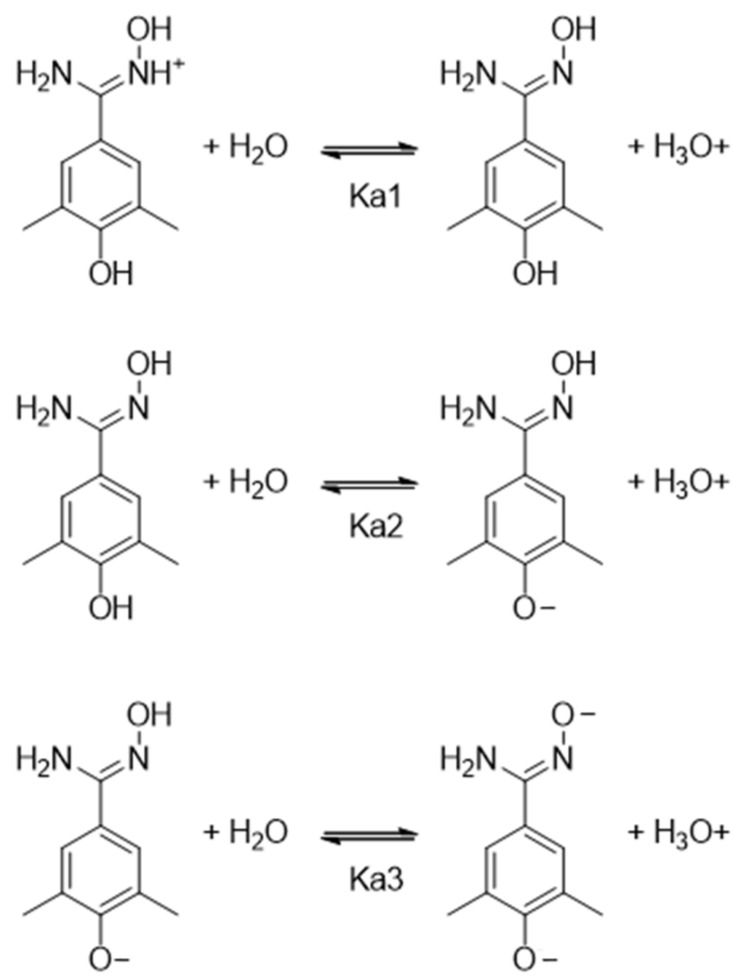
Acid-base equilibria of VIS310 (**9**).

Mehio et al. used a UV/vis titration method to measure the experimental pKa values of benzamidoxime, yielding pKa1 = 4.85 and pKa2 = 12.36 [33]. The first value (pKa1) corresponds to the deprotonated state of the protonated oxime nitrogen atom, entailing the neutral form. The second value (pKa2) is associated to the deprotonation of the oxime hydroxyl group, providing the anionic form of the compound. The presence of electron-withdrawing and/or -donating groups on the benzene ring may affect these values, entailing different stabilities of the cationic, neutral, and anionic species at a given pH. Accordingly, we used a potentiometric titration method to determine the pKa values of VIS310 and its analogues (**9**, **16**–**20**). Figure 6 shows the acid-base equilibria of VIS310 (**9**) and relative dissociation constants. 

Table 3 reports the experimental pKa values of BMS-202 (**5**), VIS310 (**9**), and its analogues (**16**–**20**), as obtained using SiriusT3 instrument. It should be mentioned that this potentiometric method can measure pKa values between 2 and 12 [34]. In general, potentiometric methods do not provide reliable data outside this range of values because of the buffering reaction of water in these pH regions (pH < 2 and pH > 12). Therefore, the very high pKa value corresponding to the deprotonation of the oxime hydroxyl group was not experimentally determined in the compounds. Conversely, one or two pKa values were obtained, depending on the presence of a phenol moiety in the benzamidoxime derivatives. In particular, VIS1200 (**16**) and VIS1204 (**20**) are characterized by one pKa value of 5.26 and 5.30, respectively. Hence, the presence of two methyl groups in the *meta* positions or a phenyl substituent in the *ortho* position exerts an electron-donating inductive effect that stabilizes the protonated nitrogen oxime. This is more evident with the insertion of the *para* hydroxyl group in the compounds VIS1201 (**17**; pKa1 = 5.35), VIS1202 (**18**; pKa1 = 5.38), VIS1203 (**19**; pKa1 = 5.38), and VIS310 (**9**; pKa1 = 5.65). These latter compounds show also a second value of the acid dissociation constant (pKa2) that corresponds to the deprotonated state of the phenolic group, entailing the anionic form of the compound. Hence, at pH 6.2 of the ligand binding experiment, the compound microspecies are mostly distributed between a cationic form and a neutral form in solution. At pH 7.2, the microspecies adopting a neutral form increases, whereas the cationic form decreases, and a minor part of the microspecies starts adopting an anionic form (Figures S2 and S3, supplementary material).

### 2.5. Binding Modes of VIS310 Analogues to PD-L1

A second round of docking studies was carried out to investigate the binding mode of VIS310 (**9**) and analogues (**16**–**20**) into the binding site of PD-L1, as detailed in the methods section. The top-scored binding mode for each compound was selected, energy refined, and visually inspected to identify intermolecular interactions. The best-scored binding modes were obtained with the cationic form of the compounds VIS310 (**9**; GScore = −7.22 kcal/mol), VIS1202 (**18**; Gscore = −6.50 kcal/mol), VIS1203 (**19**; Gscore = −6.50 kcal/mol), and VIS1201 (**17**; GScore = −6.05 kcal/mol) and with the neutral form of the compounds VIS1200 (**16**; GScore = −6.54 kcal/mol) and VIS1204 (**20**; GScore = −4.83 kcal/mol). According to the pKa determinations, both microspecies are present at pH 6.2. 

**Figure 7:**
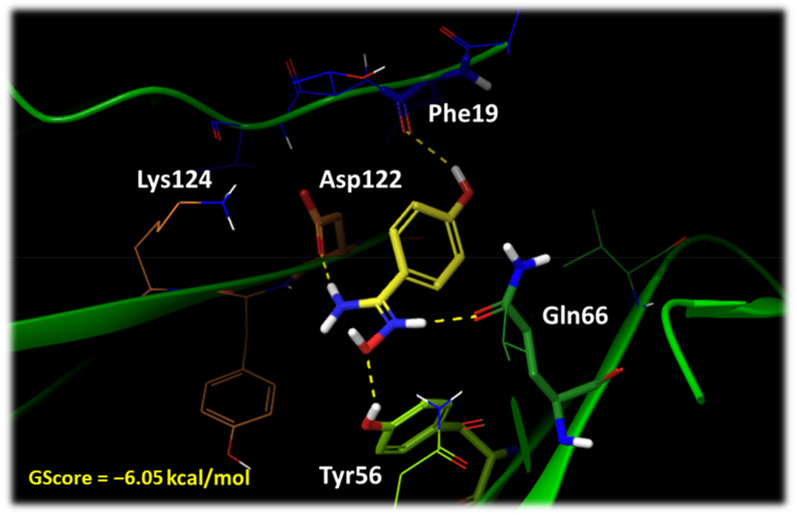
Binding mode of VIS1201 (**17**; yellow carbon atoms). Residues of PD-L1 chain C are shown with orange carbon atoms (Asp122, Lys124) and blue carbon atoms (Phe19). Residues of PD-L1 chain D are shown with green carbon atoms (Tyr56, Gln66). Hydrogen bonds are shown as yellow dashed lines.

Among the benzamidoxime derivatives, the most potent compound is VIS1201 (**17**; pH 6.2; K_d_ = 45.2 ± 10.6 μM; BEI = 28.6). The binding mode of **17** (Figure 7) shows an electrostatically enforced hydrogen bond between the positively charged oxime nitrogen atom and the side chain of Asp122 (chain C). Hydrogen bonds are established between the oxime group, the phenolic group of Tyr56 (chain D), and the amide moiety of Gln66 (chain D); a further hydrogen bond is also formed between the *para*-phenolic group of **17** and the carbonyl atom of Phe19 (chain C). 

VIS1203 (**19**) adopts a similar binding mode (Figure S4, supplementary material), though the insertion of the *ortho* methyl group does not improve the binding efficiency (**19**; pH 6.2; K_d_ = 54.9 ± 8.20 μM; BEI = 25.7). The shift of the methyl substituent in the *meta* position (VIS1202, **18**; Figure S5, supplementary material) promotes a rearrangement of the binding mode to accommodate the additional steric volume, with the positively charged oxime nitrogen of **18** now forming an electrostatic and cation-π interaction with the aromatic ring of Tyr56 (chain D) and the side chain of Asp122 (chain C), respectively. The oxime hydroxyl group makes a further hydrogen bond with the carbonyl atom of Ala121 (chain C), and the benzyl moiety engages Met115 (chain D) with hydrophobic contacts. This binding mode is also adopted by the VIS310 (**9**; Figure S6, supplementary material) that bears a methyl group in both *meta* positions of the compound. Herein, a further hydrogen bond is also observed with Gln66. The neutral form of VIS1200 (**16**; pH 6.2; K_d_ = 95.6 ± 20.6 μM; BEI = 24.5; Figure S7, supplementary material) makes a hydrogen bond between its oxime group and the side chain of Asp122 (chain C), as well as hydrophobic contacts with Met115 (chain D). In the case of VIS1204 (**20**; pH 6.2; K_d_ = 70.8 ± 12.7 μM; BEI = 19.6; Figure S8, supplementary material), it is the phenyl substituent that makes a key π-cation interaction with the side chain of Lys124 (chain C) and hydrophobic contacts with Tyr56 (chain D), whereas the oxime group forms hydrogen bonds with the backbone of Tyr123 (chain C). 

The calculated energetic scores (GScores) of the analogues do not correlate with the relative experimental dissociation constants (K_d_, Table 2). Nevertheless, the proposed binding modes agree with the above SAR scheme of VIS310 analogues, suggesting the relevance of the hydroxyl group in the *para* position of the protonated benzamidoxime scaffold. Indeed, its presence affects the number of interactions with PD-L1 (e.g., VIS1201 and VIS1203). Moreover, the detrimental effect of the methyl substituents in the *ortho* or *meta* position on the binding efficiency (BEI) is also evidenced by the binding modes of the relative compounds, with such groups not being involved in specific contacts with hydrophobic residues. Overall, this is in line with previous works suggesting that scoring functions are generally more capable of identifying the correct ligand binding poses than predicting binding affinities [35,36,37].

### 2.6. Ligand Binding Displacement Assay of PD-1/PD-L1 Complex

In this part of the study, a ligand binding displacement assay was designed to assess the ability of the most active PD-L1 ligand, namely, VIS1201 (**17**), to block the formation of the PD-1/PD-L1 complex. This study was carried out with MST.

**Figure 8:**
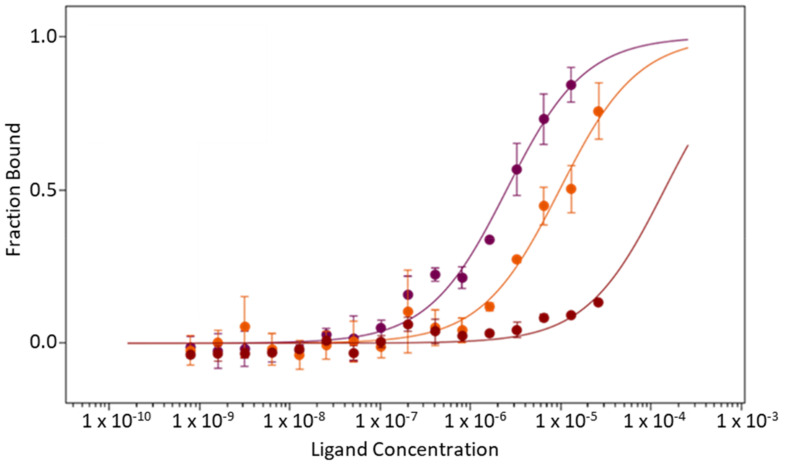
MST binding curves of PD-1 to NT650/PD-L1 at pH 6.2 (purple circles and line) and in presence of 50 µM VIS1201 (orange circles and line), 100 µM VIS1201 (red circles and line).

As a first experiment, the dissociation constant of PD-1 to NT650/PD-L1 was assessed at pH 6.2, yielding a K_d_ value of 2.50 ± 0.50 μM. Next, PD-1 binding experiments to NT650/PD-L1 were performed in the presence of increasing concentrations of VIS1201 (**17**) (50 μM and 100 μM) to monitor the impact of such a compound on the formation of the complex, as measured by the shift of the K_d_ value. It was found that VIS1201 (**17**) dose-dependently inhibits the PD-1/PD-L1 interaction, with the K_d_ value of the immune checkpoint complex shifting to poorer values (Table 4 and Figure 8).

## 3. Discussion

The inhibition of PD-1/PD-L1 complex formation is pursued in drug discovery for expanding therapeutic strategies in immuno-oncology. Although several mAbs against both PD-1 and PD-L1 have been advanced in clinical use, these biologic drugs suffer from a narrow therapeutic response in cancer patients. Hence, the quest is still active for drug-like small molecules with wider clinical efficacy that may also overcome the limited bioavailability, high production costs, and safety issues of biologic drugs. 

The pH of the tumor microenvironment is known to be altered in many types of cancer upon lactic acid production [25,26], including pancreatic cancer. In particular, the microenvironment of pancreatic cancer is characterized by a low extracellular pH that, along with nutrient deprivation and hypoxia conditions, leads to tumor cell survival and resistance to therapy, promoting tumor invasion and metastasis [30]. Targeting cancer cells with pH-sensitive drugs may thus represent a promising strategy to improve the efficacy of cancer therapy.

In a previous study, we found that alterations of pH, as those occurring in the tumor microenvironment, affect the strength of ligand binding to PD-L1 [27]. That study highlighted the relevance of electrostatic force in ligand/protein interactions of PD-1/PD-L1 inhibitors. 

Leveraging on this finding, herein, we have performed a structure-based screening campaign that has led to identifying VIS310 (**9**) as a hit compound with pH-dependent binding potency. This compound bears a benzamidoxime scaffold that has been instrumental to running an analogue-based screening campaign for enabling early optimization efforts of PD-L1 binding activity. The identified benzamidoxime derivatives (**9**, **16**–**20**) have different pKa values as a result of the electron-donor effects of the functional groups that stabilize the protonated nitrogen oxime and contribute to the dissociation constants (K_d_) that have been obtained in ligand binding experiments with MST at a pH of 6.2 and 7.2. 

The pH-dependent binding potency of these compounds to PD-L1 is evidenced when comparing the shifts of BEI at both tested pH values with respect to BMS-202 (**5**). Indeed, this latter compound shows a delta increment of only +0.7 (**5**; pH 7.2 BEI = 12.1; pH 6.2 BEI = 12.8), suggesting a marginal pH-dependent binding potency that agrees with the presence of a biphenyl moiety and its hydrophobic interactions as a major contributing force to the compound’s binding efficiency. Conversely, the delta increment of BEI is larger in VIS310 (**9**) analogues, ranging from +1.5 (**20**; pH 7.2 BEI = 18.1; pH 6.2 BEI = 19.6) to +8.9 (**17**; pH 7.2 BEI = 19.7; pH 6.2 BEI = 28.6). The significant pH-dependent increment of BEI may represent an opportunity of benzamidoxime derivatives to achieve a specific targeting of cancer cells expressing PD-L1 in the acidic tumor microenvironment, thus reducing the risk of iRAEs associated to a wider targeting against other cells expressing PD-L1.

A ligand binding displacement assay with MST has proven the ability of the most potent benzamidoxime derivative, namely, VIS1201 (**17**), to dose-dependently inhibit the formation of the PD-1/PD-L1 complex. It is the high BEI of compound **17** (BEI = 28.6) that may likely account for the ability to compete with PD-1 for the binding interaction to PD-L1, with the compound exploiting electrostatically enforced hydrogen bonds with Asp122 of PD-L1 as a major driving force.

In conclusion, VIS1201 (**17**) is a novel inhibitor of the PD-1/PD-L1 interaction that is endowed with a pH-dependent binding potency and a better BEI than BMS-202 (**5**). The obtained preliminary structure–activity relationships (SARs), as well as the proposed binding modes, support further efforts of optimization cycles of benzamidoxime derivatives as a novel chemical class of PD-L1 ligands with pH-dependent binding potency. These efforts will prove useful for the design of next-generation small molecule inhibitors of PD-L1, with tailored physicochemical properties for making them resilient to drug-resistance mechanisms based on the acidification of the tumor microenvironment, as well as endowed with an improved safety profile.

## 4. Materials and Methods

*Structure-based Screening and Analogue-Based Screening*. The aim of the structure-based screening campaign was the identification of small molecules with pH-dependent binding activity to PD-L1. To achieve this goal, a virtual library of small molecules was docked into a binding site of PD-L1 defined around the residues Asp122 and Lys124 (Figure 2), which were identified as key residues (hot-spots) for ligand recognition in a previous work [27]. 

Accordingly, a virtual library of 5801 small molecules was assembled, merging an in-house collection of readily available compounds with MW ≤ 500 dalton (807 molecules) with an external collection of highly soluble fragments (4994 molecules) downloaded from Life Chemicals Inc. in February 2022. The chemical structures of these compounds were drawn and refined using LigPrep (Schrödinger Release 2021-3; LigPrep, Schrödinger, LLC, New York, NY, USA), adding hydrogens, generating all ionization and tautomeric states at pH = 7 ± 2, and optimizing their geometry with the OPLS4 force field. No compound was discarded after such a refinement process.

The crystal structure of PD-L1 bound to BMS-202 (PDB code 5J89) [24] was downloaded from Research Collaboratory for Structural Bioinformatics (RCSB) PDB (www.rcsb.org) [38]. Chains C/D were kept with the co-crystallized ligand **5** bound into the cavity shaped by the GFC surface of the two PD-L1 units (Figure 2). The Protein Preparation Wizard (PPW) tool implemented in Maestro (Schrödinger Release 2021-3; Maestro, Schrödinger, LLC, New York, NY, USA) was used to process and energetically refine the chain C/D structures, removing water and detergent molecules, adding hydrogen atoms, and optimizing the internal geometries. The OPLS4 force field was used with an atomic coordinate displacement restrain on heavy atoms set to 0.30 Å. 

Docking studies of the chemical library into the binding site shaped by chains C and D of 5J89 were performed employing Glide (Schrödinger Release 2021-3: Glide, Schrödinger, LLC, New York, NY, USA) [39]. The grid box was defined with its center located close to Asp122 and Lys124 of chain C (x = 16.21, y = 31.30, z = 186.51). The inner grid box was sized 10 × 10 × 10 Å. Docking studies were carried out using the standard precision (SP) method and the GScore scoring function (kcal/mol). The criteria for selection of the initial pose were set at the default values, keeping n.5000 poses per ligand for the initial phase of docking within a scoring window of 100 kcal/mol, and keeping best n.400 poses per ligand for energy minimization with no use of expanded sampling. 

Compounds of the library showing a GScore lower than −4 kcal/mol, as well as engaging Asp122 and/or Lys124 with electrostatic and/or hydrogen bond interactions, were short-listed. Specifically, the energy cutoff of −4 kcal/mol was chosen on the basis of previous data suggesting this value as a good arbitrary threshold to identify hit compounds amid successfully docked ligands [37]. However, it should be stressed that GScore values do not generally correlate with the experimental binding data [35,36,37], pinpointing that further work is required to improve such a scoring function for reducing the number of false-positive virtual hits and/or the discard of active compounds. The presence of polar interactions with Asp122 and/or Lys124 was sought as a key feature of the selected hit compounds to increase the likelihood of having pH-dependent binding activity.

A first group of virtual hit compounds was selected picking the top-scored 50 readily available compounds. A second list of virtual hit compounds was selected picking the top-scored 10 highly soluble fragments by taking into account their structural diversity from the first group of selected compounds. Specifically, the structural diversity between the two groups of selected compounds was confirmed by performing a multidimensional scaling analysis (MDS, similarity cutoff = 0.4) on ChemMine, using atom pair similarity measures and transforming the generated Tanimoto coefficients into distance values for the MDS analysis (Figure S1, supplementary material) [32]. The chemical structures (smile codes) of all selected compounds are listed in Table S1, supplementary material.

The analogue-based screening campaign was performed with the aim of identifying structural analogues of VIS310 (**9**) for developing structure–activity relationships that could be instrumental for future medicinal chemistry efforts of lead optimization. It was carried out using the benzamidoxime scaffold of VIS310 (**9**) as the query substructure. The assembled virtual library of 5801 small molecules and the REAL database (about 43.8 million drug-like compounds) of Enamine (Riga, Latvia) were used as sources of the quest for VIS310 analogues (**16**–**20**).

*Docking studies of hit compounds*. Docking studies of VIS310 (**9**) and its analogues (**16**–**20**) were carried out using Glide (Schrödinger Release 2021-3: Glide, Schrödinger, LLC, New York, NY, USA) and the grid box in the structure of PD-L1 as defined for the structure-based screening campaign. The standard precision (SP) method and the GScore scoring function (kcal/mol) were used in combination with specific criteria for the selection of the initial pose: (i) keeping n.50,000 poses per ligand for the initial phase of docking; (ii) using a scoring window of 500 kcal/mol; (iii) keeping the best n.1000 poses per ligand for energy minimization; (iv) using the expanded sampling mode. A good performance of this docking protocol has been reported in the literature to predict the binding mode of fragment hit compounds [36]. The top-scored docking pose for each molecule was then selected and submitted to a further cycle of energy minimization to refine the intermolecular interactions between the ligand and target protein. The energy minimization was carried out using the OPLS4 force field with the Polak–Ribière conjugate gradient (PRCG) method [40], until a gradient convergence threshold criteria of 0.05 kJ mol^−1^ Å^−1^ was reached for all complexes. 

*Biophysical Binding Assays*. A lyophilized form of the recombinant human PD-L1 protein was purchased from Abcam (ab167713, Abcam, Cambridge, UK) and reconstituted by adding sterile deionized water to reach a theoretical concentration of 400 µg/mL in PBS (PBS7.2: 50 mM KH_2_PO_4_, 0.05% Tween 20, pH 7.2). The effective protein concentration was determined by assessing the absorbance at 205 nm (Ɛ_205nm_ = 31 M^−1^ cm^−1^) [41] of the reconstituted sample with a NanoDrop-One spectrophotometer, yielding a concentration of 12 µM. Then, the protein was labeled with a fluorescent dye using the Protein Labelling Kit RED-NHS (NT650, NanoTemper Technologies GmbH, Munich, Germany) and a 1:4 ratio (protein:dye). For the labeling reaction, a volume of 100 µL of 12 µM solution of protein in phosphate buffer PBS7.2 was mixed with 100 µL of 48 µM NT650 fluorophore and incubated for 30 min at room temperature (RT) in the dark. The unbounded fluorophore was removed by a size-exclusion chromatography column using a PBS7.2 storage buffer (pH 7.2). The removal of the unbounded fluorophore was also repeated by conditioning the size-exclusion chromatography column with a phosphate buffer at pH 6.2 (PBS6.2: 50 mM KH_2_PO_4_, 0.05% Tween 20, pH 6.2). The concentration of the labeled protein (NT650/PD-L1) in both storage buffers at pH 7.2 and 6.2 was determined by absorption spectroscopy, yielding a degree of labeling (DoL) of 0.54 in both cases.

The stability of the NT650/PD-L1 protein at pH 7.2 and 6.2 was checked using label-free thermal shift analysis with a Tycho instrument (NT.6; NanoTemper Technologies GmbH, Munich, Germany). Specifically, label-free thermal shift analysis monitors changes in the emission intensity and wavelength maximum of intrinsic fluorescence properties of buried tryptophane and tyrosine residues that become exposed in the unfolded state of the protein upon an increasing temperature from 35 °C to 95 °C. The recombinant PD-L1 (wt/PD-L1) and labeled NT650/PD-L1 proteins were diluted in the relative PBS (PBS7.2, PBS6.2) at a concentration of 2.7 µM. Samples were loaded into Tycho NT.6 capillaries, and the thermal unfolding profiles of the protein samples were recorded for comparative analysis in three independent experiments. Inflection temperature (T_i_) values are reported as mean ± standard deviation values (Table S2, supplementary material). No shift of the inflection temperature (ΔT_i_ < 1.0) was observed between unlabeled wt/PD-L1 and NT650/PD-L1 at pH 7.2 and 6.2, suggesting that both the labeling process and the different pH conditions do not affect the stability of the protein.

Ligand binding experiments were performed using MST (Monolith NT.115; NanoTemper Technologies GmbH, Munich, Germany). This method is based on the principle of thermophoresis, which is the movement of a biomolecular complex in a temperature gradient depending on the size, charge, and hydration shell that typically change upon ligand/target interaction [42]. The selected 10 highly soluble fragments were purchased from Life Chemicals. BMS-202 (**5**) was purchased from Selleckchem (Selleckchem, Houston, DX, USA) and used as positive control. A vehicle of DMSO 2% was used as negative control. 

Single-point binding assays were carried out, recording the MST signal for each capillary at medium laser power and 40% LED power, choosing the manual mode with a 19/20 s hot region. Each experiment was repeated three times employing premium-coated capillaries. Compounds were tested at a 250 μM concentration against 50 nM NT650/PD-L1, using a diluted mixture with 2% DMSO in PBS7.2. As a rule of thumb, compounds were defined as binders when producing a fluorescent signal (F-norm) outside the signal value and three-fold standard deviations of the vehicle (F-norm = 760 ± 3 × 3.5).

Full-point binding assays were carried out on binders identified in the single-point assay to determine the dissociation constant (K_d_) values against NT650/PD-L1 at pH 7.2 and 6.2. Each compound was tested starting the 1:1 dilution from a 1 mM concentration in MST buffer (PBS7.2 and PBS6.2) with 2% DMSO. An amount of 10 µL solution of 100 nM NT650/PD-L1 was added to each compound dilution and mixed to halve the final concentration of each component, including 2% DMSO, and reach a reaction volume of 20 µL. After incubating the mixtures for 30 min, samples were loaded into 16 premium-coated capillary tubes for MST analysis. MST signals were recorded for each capillary at medium laser power and 40% LED power, choosing the manual mode with 19/20s hot region. 

The Binding Efficiency Index (BEI) was calculated using the following equation [Equation (1)] [43]:

BEI = pK_d_/MW (kDa) (1)

Ligand binding displacement assays were carried out with MST. The recombinant extracellular domain of human PD-1 was purchased from Abcam (ab174035, Abcam, Cambridge, UK) in lyophilized form. The reconstitution of the protein was done by adding sterile deionized water to achieve a final concentration of 1 mg/mL in PBS7.2. The MST binding experiments of PD-1 were carried out against NT650/PD-L1 in PBS6.2 with 5% glycerol and 2% DMSO. The protein was tested starting the 1:1 dilution from a 26 μM concentration in MST buffer (PBS6.2) with 2% DMSO. An amount of 10 µL solution of 20 nM NT650/PD-L1 was added to PD-1 protein dilution and mixed to achieve a final protein concentration of 10 nM, 5% glycerol, and a reaction volume of 20 μL. After incubating the mixtures for 40 min in the dark, samples were loaded into 16 premium-coated capillary tubes for MST analysis. MST signals were recorded for each capillary at medium laser power and 80% LED power, choosing the Default on Time (DoT) mode with 4/5s hot region. 

Binding experiments of PD-1 were repeated after incubating NT650/PD-L1 with VIS1201 (**17**) for 15 min at a concentration corresponding to its K_d_ value (50 μM) and twice this value (100 μM). The mixture containing NT650/PD-L1 and VIS1201 (**17**) was added to PD-1 pre-dilutions, following the above MST protocol of the PD-1 binding experiments. 

In all binding experiments and ligand binding displacement assays, raw data were analyzed using MO.Affinity analysis software v2.3 (NanoTemper Technologies GmbH, Munich, Germany). K_d_ values were extrapolated by compound concentration-dependent changes of the NT650/PD-L1 F-norm signals using MO.Affinity analysis software v2.3. 

Confidence values (±) are reported next to K_d_ values, indicating the range where the K_d_ falls with 68% of certainty. The Signal-to-Noise ratio (S/N) was >12 in all MST experiments, indicating excellent assay conditions. MST experiments were carried out using a Monolith NT.115 instrument (NanoTemper Technologies GmbH, Munich, Germany).

*Physicochemical Property Evaluations*. Acidic constants (_calc_pKa) of compounds were calculated using the calculator plugin Marvin (v20.11, 2021; ChemAxon, Budapest, Hungary). Experimental pKa values (pKa) were determined within the range of pKa 2–12 at 25 °C, using the Sirius T3 potentiometric method (Pion Inc, E. Sussex, UK) [34]. 

Each experiment was performed by specifying the sample weight, number of expected pKa values, calculated pKa (_calc_pKa) values, titration mode, and number of titrations (n.3). In order to measure the pKa of a compound, a sample concentration of 1 mM was prepared and dissolved, adding methanol as the co-solvent. Titrations of VIS310 (**9**) were performed at different methanol/water ratios (50% methanol, 40% methanol, and 30% methanol) to determine the aqueous pKa. Titrations of compounds **16**–**20** were performed using a ratio of 45%, 40%, 35% methanol. The Yasuda–Shedlovsky method was chosen to extrapolate aqueous pKa values. The R^2^ value was used to evaluate the validity of the assay with a co-solvent, and the results were considered acceptable with a R^2^ value ≥ 0.9 (Table S3, supplementary material). The accuracy of the pKa assay was also evaluated using the buffer index profile of the analyte. Specifically, the buffering capacity signal of the analyte must be above the intrinsic buffering capacity of water in order to have an accurate potentiometric measurement. Results were analyzed using Sirius T3 software v1.1.3.

## Figures and Tables

**Table 1:** Binding energy score (GScore), single-point binder flag, dissociation constants (K_d_), molecular weight (MW), and binding efficiency index (BEI) of BMS-202 (**5**) and putative hit compounds **6**–**15** against PD-L1.

Virtual Hits	GScore (kcal/mol)	Binder ^[a]^	K_d_ ± c.v. ^[b]^ (μM)	MW (Dalton)	BEI
BMS-202 (**5**)	−10.22	x	8.13 ± 1.38	419	12.1
VIS284 (**6**)	−7.94	x	>250	192	n.d. ^[c]^
VIS285 (**7**)	−7.05	x/o	>250	175	n.d.
VIS303 (**8**)	−9.13	x	>250	299	n.d.
VIS310 (**9**)	−7.22	x	163.75 ± 33.61	180	21.0
VIS545 (**10**)	−9.09	x/o	>250	269	n.d.
VIS826 (**11**)	−6.05	x	>250	316	n.d.
VIS877 (**12**)	−4.76	x	>250	226	n.d.
VIS879 (**13**)	−4.56	x	>250	254	n.d.
VIS1079 (**14**)	−7.61	x/o	>250	209	n.d.
VIS32926 (**15**)	−7.71	x/o	>250	195	n.d.

^[a]^ x: binder, x/o: borderline binder; ^[b]^ c.v.: confidence values calculated on n.3 independent experiments; ^[c]^ n.d.: not determined.

**Table 2:** Dissociation constants (K_d_), molecular weight (MW), and binding efficiency index (BEI) of BMS-202 (**5**), VIS310 (**9**), and analogues **16**–**20** against PD-L1 at pH 6.2 and 7.2.

Virtual Hits	pH 7.2 K_d_ ± c.v. ^[a]^ (μM)	pH 6.2 K_d_ ± c.v. ^[a]^ (μM)	MW (Dalton)	BEI (pH 6.2)
BMS-202 (**5**)	8.13 ± 1.38	4.50 ± 0.56	419	12.8
VIS310 (**9**)	164 ± 33.6	47.8 ± 11.5	180	24.0
VIS1200 (**16**)	>1000	95.6 ± 20.6	164	24.5
VIS1201 (**17**)	>1000	45.2 ± 10.6	152	28.6
VIS1202 (**18**)	>1000	55.4 ± 9.86	166	25.6
VIS1203 (**19**)	>1000	54.9 ± 8.20	166	25.7
VIS1204 (**20**)	148 ± 71.3	70.8 ± 12.7	212	19.6

^[a]^ c.v.: confidence values calculated on n.3 independent experiments.

**Table 3:** Acid dissociation constants (pKa) of BMS-202 (**5**), VIS310 (**9**), and analogues (**16**–**20**).

Compounds	pKa1	pKa2	pKa3
BMS-202 (**5**)	n.d. ^[a]^	8.39 ± 0.05 ^[c]^	-
VIS310 (**9**)	5.65 ± 0.01 ^[b]^	9.32 ± 0.02 ^[d]^	n.d. ^[e]^
VIS1200 (**16**)	5.26 ± 0.01 ^[b]^	-	n.d. ^[e]^
VIS1201 (**17**)	5.35 ± 0.12 ^[b]^	8.64 ± 0.01 ^[d]^	n.d. ^[e]^
VIS1202 (**18**)	5.38 ± 0.01 ^[b]^	8.97 ± 0.03 ^[d]^	n.d. ^[e]^
VIS1203 (**19**)	5.38 ± 0.01 ^[b]^	9.17 ± 0.02 ^[d]^	n.d. ^[e]^
VIS1204 (**20**)	5.30 ± 0.01 ^[b]^	-	n.d. ^[e]^

^[a]^ pKa1: pyridine nitrogen atom (**5**)**,** n.d.: not determined (pKa < 2, out of the range of values of SiriusT3); ^[b]^ pKa1: oxime nitrogen atom (**9**, **16**–**20**); ^[c]^ pKa2: secondary amine group (**5**); ^[d]^ pKa2: phenolic group (**9**, **17**–**19**); ^[e]^ pKa3: oxime hydroxyl group (**9**, **16**–**20**), n.d.: not determined (pKa > 12, out of the range of values of SiriusT3). All pKa values are reported as mean ± standard deviations of n.3 titration experiments.

**Table 4:** Dissociation constants (K_d_) of PD-1/PD-L1 in presence of increasing concentrations of VIS1201 (**17**) at pH 6.2.

	Vehicle K_d_ ± c.v. ^[a]^ (pH 6.2)	VIS1201 (50 μM) K_d_ ± c.v. ^[a]^ (pH 6.2)	VIS1201 (100 μM) K_d_ ± c.v. ^[a]^ (pH 6.2)
PD-1/PD-L1	2.50 ± 0.50	9.68 ± 2.57	>30

^[a]^ c.v.: confidence values calculated on n.3 independent experiments.

## Data Availability

Not applicable.

## Supplementary Materials

The following supporting information can be downloaded at: https://www.mdpi.com/article/10.3390/ijms24065535/s1.
