# Supplementary material for: Turning a Tumor Microenvironment Pitfall into Opportunity: Discovery of Benzamidoxime as PD-L1 Ligand with pH-Dependent Potency
Source: Int J Mol Sci. 2023 Mar 14;24(6):5535. doi: 10.3390/ijms24065535 (PMC10054428; doi:10.3390/ijms24065535)
Supplement: Supplementary file 1 [file ijms-24-05535-s001.zip › ijms-2272165-supplementary.pdf]

Table of Contents:

Figure S1. Plot of the distance values (V1 and V2) resulting from the MDS analysis with ChemMine. Selected highly soluble fragments are red labelled (VIS1075-VIS1084). The confirmed hit compound (VIS310) is also labelled.

Figure S2. Molar fraction of VIS310 (**9**) microspecies as a function of pH. Relative abundancies of microspecies at pH 6.2 and 7.2 are reported at the bottom of the graph.

Figure S3. Molar fraction of VIS1201 (**17**) microspecies as function of pH. Relative abundancies of microspecies at pH 6.2 and 7.2 are reported at the bottom of the graph.

Figure S4. Binding mode of VIS1203 (**19**; yellow carbon atoms) to PD-L1. Residues of PD-L1 chain C are shown with orange carbon atoms (Asp122, Lys124) and blue carbon atoms (Phe19). Residues of PD-L1 chain D are shown with green carbon atoms (Tyr56, Gln66). Hydrogen bonds are shown as yellow dashed lines. Electrostatic interactions are shown as magenta dashed lines.

Figure S5. Binding mode of VIS1202 (**18**; yellow carbon atoms) to PD-L1. Residues of PD-L1 chain C are shown with orange carbon atoms (Ala121, Asp122) and blue carbon atoms (Phe19). Residues of PD-L1 chain D are shown with green carbon atoms (Tyr56) and orange carbon atoms (Met115). Hydrogen bonds are shown as yellow dashed lines. Cation-π interactions are shown as green dashed lines.

Figure S6. Binding mode of VIS310 (**9**; yellow carbon atoms) to PD-L1. Residues of PD-L1 chain C are shown with orange carbon atoms (Ala121, Asp122) and blue carbon atoms (Phe19). Residues of PD-L1 chain D are shown with green carbon atoms (Tyr56, Gln66) and orange carbon atoms (Met115). Hydrogen bonds are shown as yellow dashed lines. Cation-π interactions are shown as green dashed lines.

Figure S7. Binding mode of VIS1200 (**16**; yellow carbon atoms) to PD-L1. Residues of PD-L1 chain C are shown with orange carbon atoms (Asp122, Lys124). Residues of PD-L1 chain D are shown with green carbon atoms and orange carbon atoms (Met115). Hydrogen bonds are shown as yellow dashed lines.

Figure S8. Binding mode of VIS1204 (**20**; yellow carbon atoms) to PD-L1. Residues of PD-L1 chain C are shown with orange carbon atoms (Asp122, Lys124, Tyr123). Residues of PD-L1 chain D are shown with green carbon atoms (Tyr56). Hydrogen bonds are shown as yellow dashed lines. Cation-π interactions are shown as green dashed lines.

Table S1. Virtual hit compounds with chemical structure (smile format), molecular weight (MW, Dalton) and binding energy scores (Score, kcal/mol). Compounds VIS1075-VIS1084 belong to the subset of highly soluble fragments. Remaining compounds are from the internal subset of readily available small molecules. Red typed entries indicate putative hit compounds according to single-point binding assays.

Table S2. Inflection temperature (T_i), initial ratio and differential (Δ) ratio of wt-PD-L1 and labelled NT650/PD-L1 at pH 7.2 and 6.2.

Table S3. R^2 values of the pKa assays according to the Yasuda-Shedlovsky method.

Figure S1. Plot of the distance values (V1 and V2) resulting from the MDS analysis with ChemMine. Selected highly soluble fragments are red labelled (VIS1075-VIS1084). The confirmed hit compound (VIS310) is also labelled.

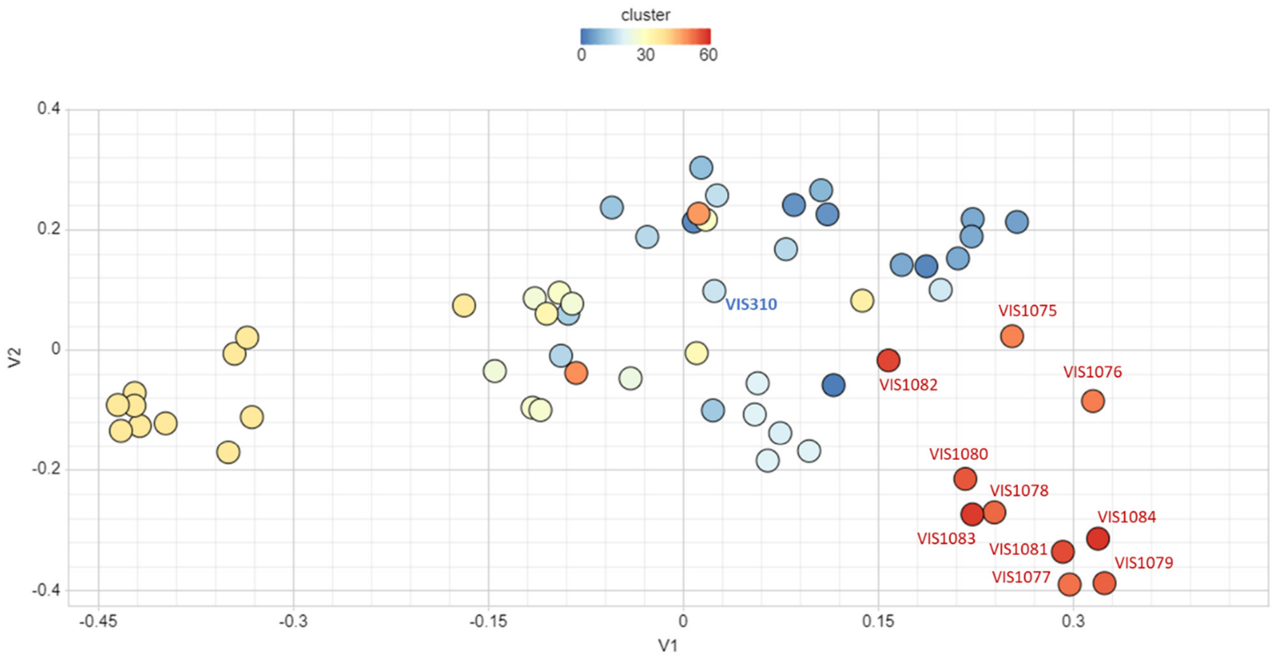

Figure S2. Molar fraction of VIS310 (**9**) microspecies as a function of pH. Relative abundancies of microspecies at pH 6.2 and 7.2 are reported at the bottom of the graph.

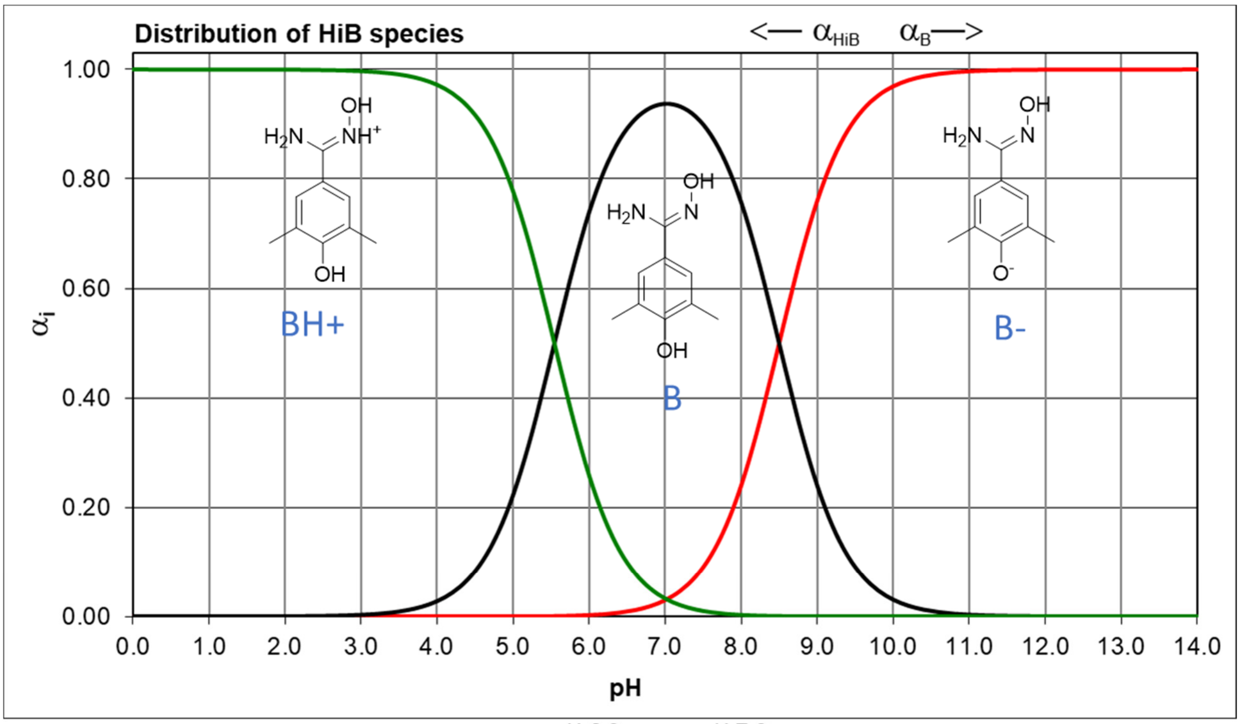

pH 6.2	pH 7.2
BH+ = 0.180	BH+ = 0.021
B = 0.818	B = 0.963
B- = 0.001	B- = 0.015

Figure S3. Molar fraction of VIS1201 (**17**) microspecies as function of pH. Relative abundancies of microspecies at pH 6.2 and 7.2 are reported at the bottom of the graph.

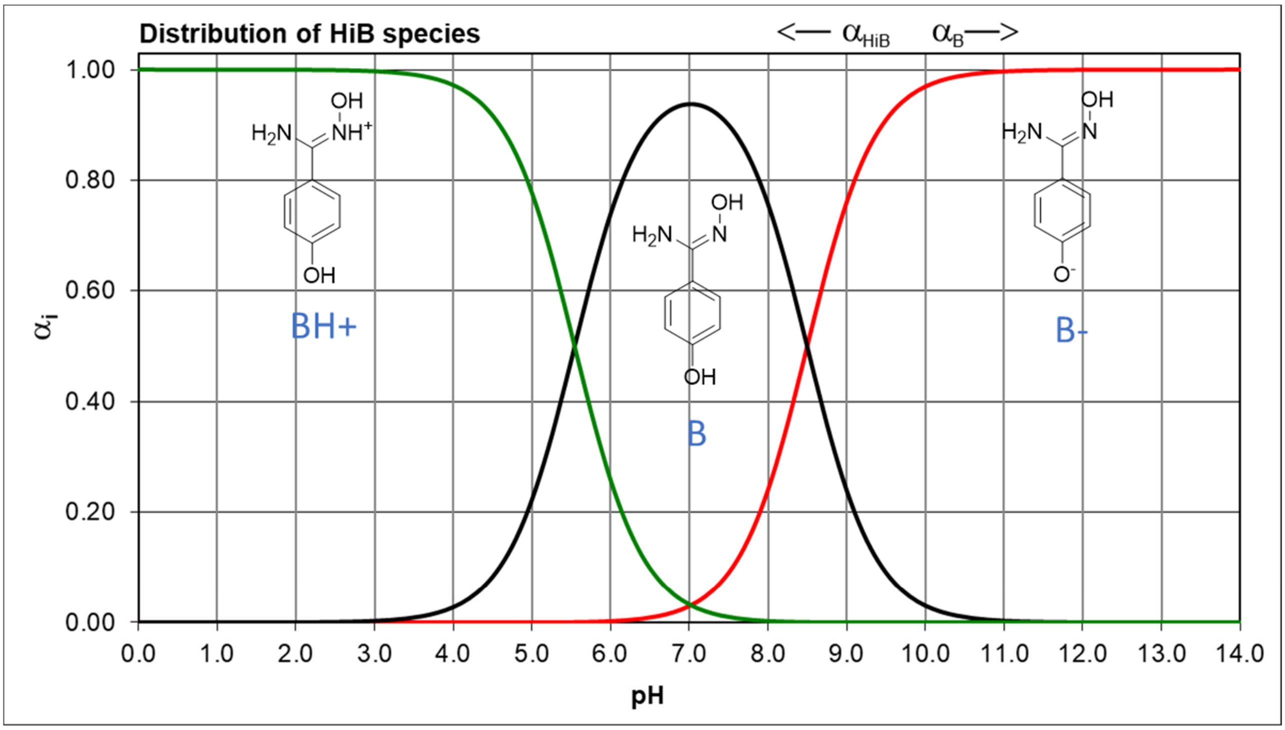

pH 6.2	pH 7.2
BH+ = 0.099	BH+ = 0.010
B = 0.894	B = 0.920
B- = 0.007	B- = 0.070

Figure S4. Binding mode of VIS1203 (**19**; yellow carbon atoms) to PD-L1. Residues of PD-L1 chain C are shown with orange carbon atoms (Asp122, Lys124) and blue carbon atoms (Phe19). Residues of PD-L1 chain D are shown with green carbon atoms (Tyr56, Gln66). Hydrogen bonds are shown as yellow dashed lines. Electrostatic interactions are shown as magenta dashed lines.

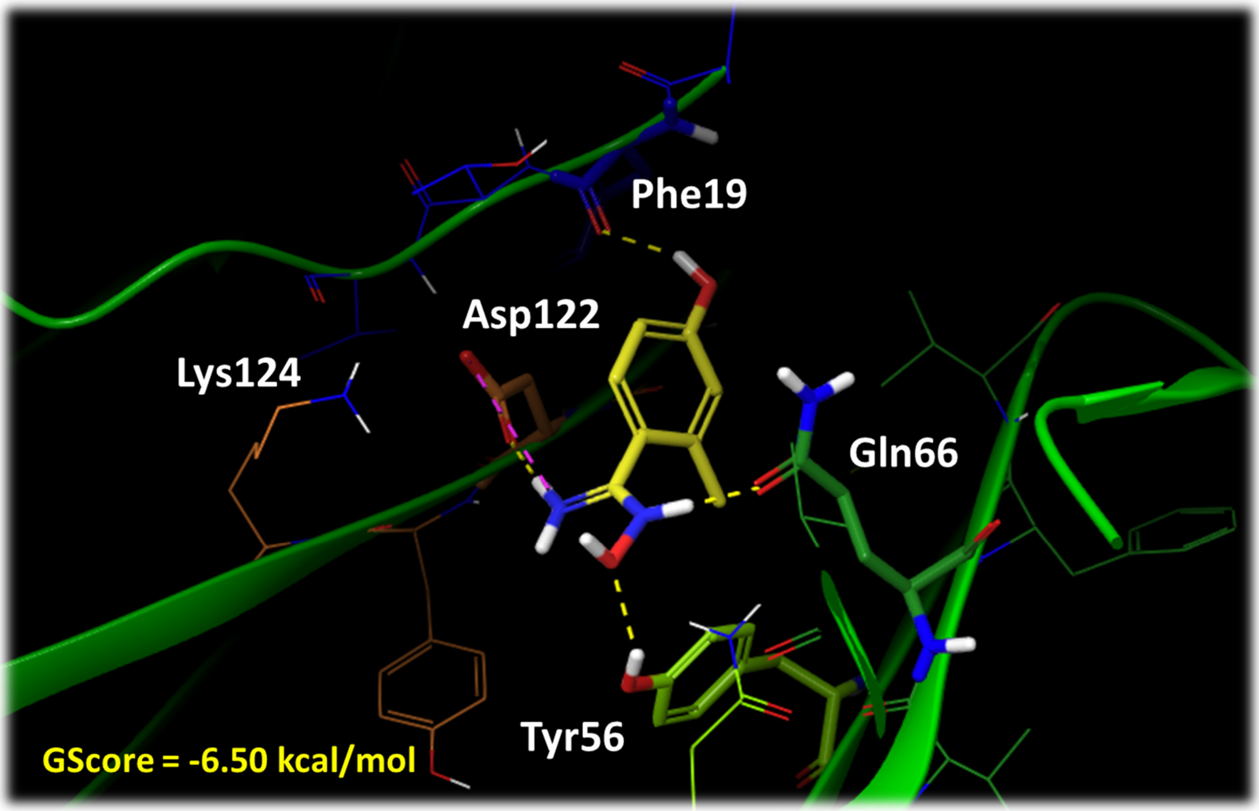

Figure S5. Binding mode of VIS1202 (**18**; yellow carbon atoms) to PD-L1. Residues of PD-L1 chain C are shown with orange carbon atoms (Ala121, Asp122) and blue carbon atoms (Phe19). Residues of PD-L1 chain D are shown with green carbon atoms (Tyr56) and orange carbon atoms (Met115). Hydrogen bonds are shown as yellow dashed lines. Cation-π interactions are shown as green dashed lines.

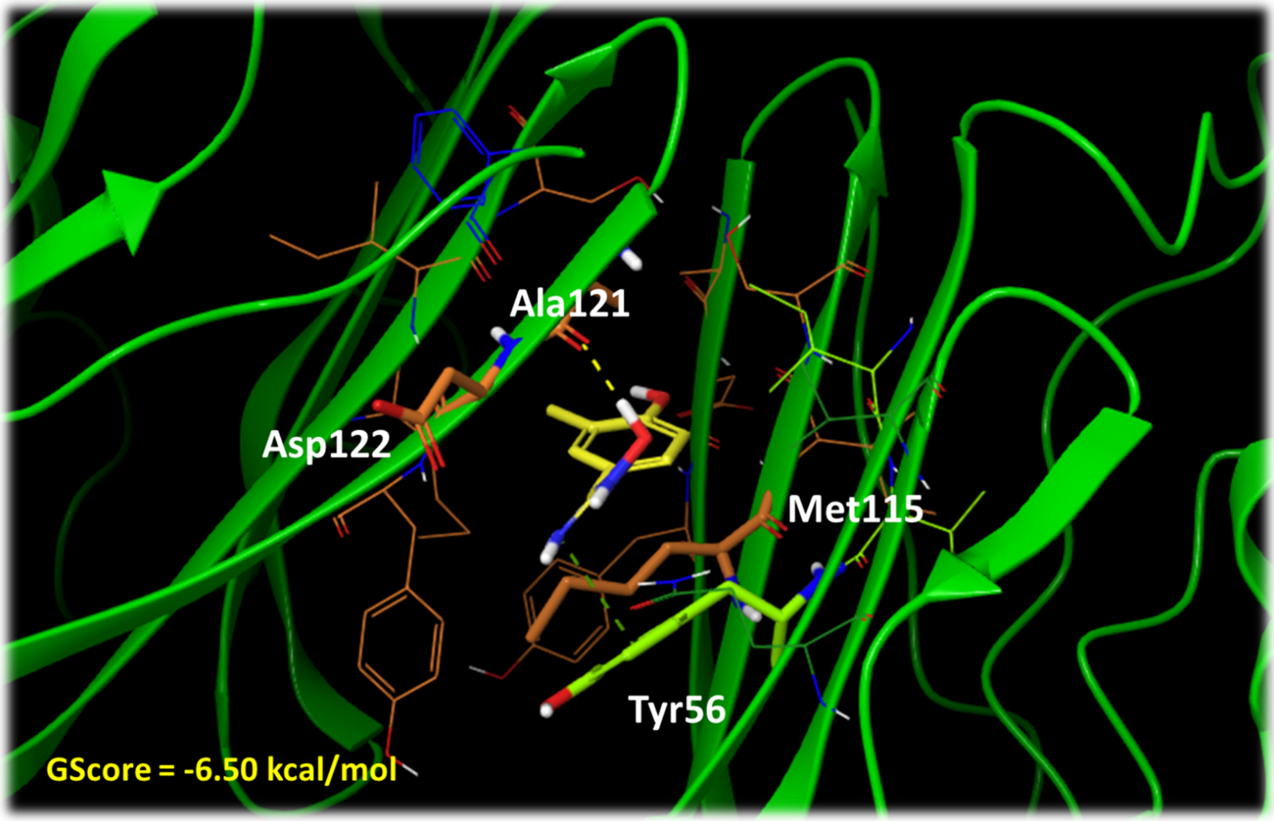

Figure S6. Binding mode of VIS310 (**9**; yellow carbon atoms) to PD-L1. Residues of PD-L1 chain C are shown with orange carbon atoms (Ala121, Asp122) and blue carbon atoms (Phe19). Residues of PD-L1 chain D are shown with green carbon atoms (Tyr56, Gln66) and orange carbon atoms (Met115). Hydrogen bonds are shown as yellow dashed lines. Cation-π interactions are shown as green dashed lines.

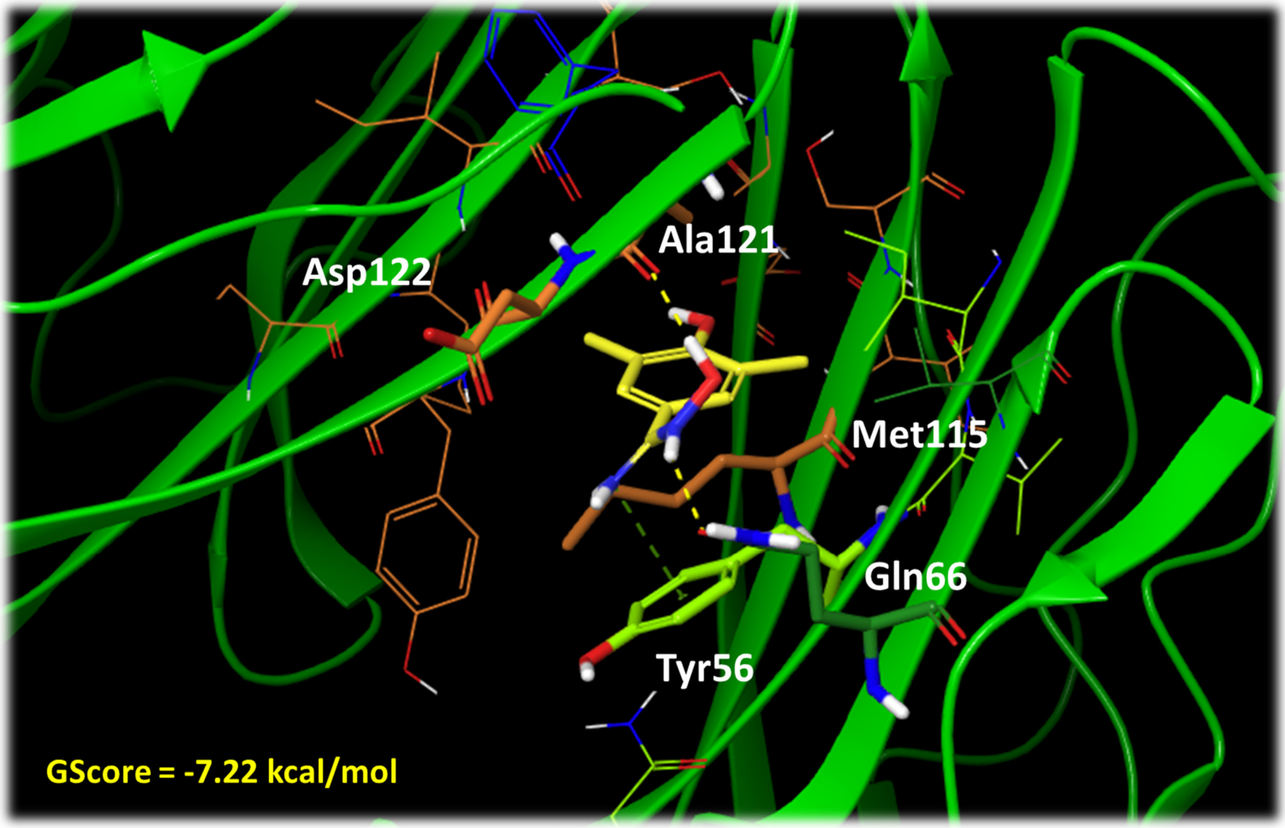

Figure S7. Binding mode of VIS1200 (**16**; yellow carbon atoms) to PD-L1. Residues of PD-L1 chain C are shown with orange carbon atoms (Asp122, Lys124). Residues of PD-L1 chain D are shown with green carbon atoms and orange carbon atoms (Met115). Hydrogen bonds are shown as yellow dashed lines.

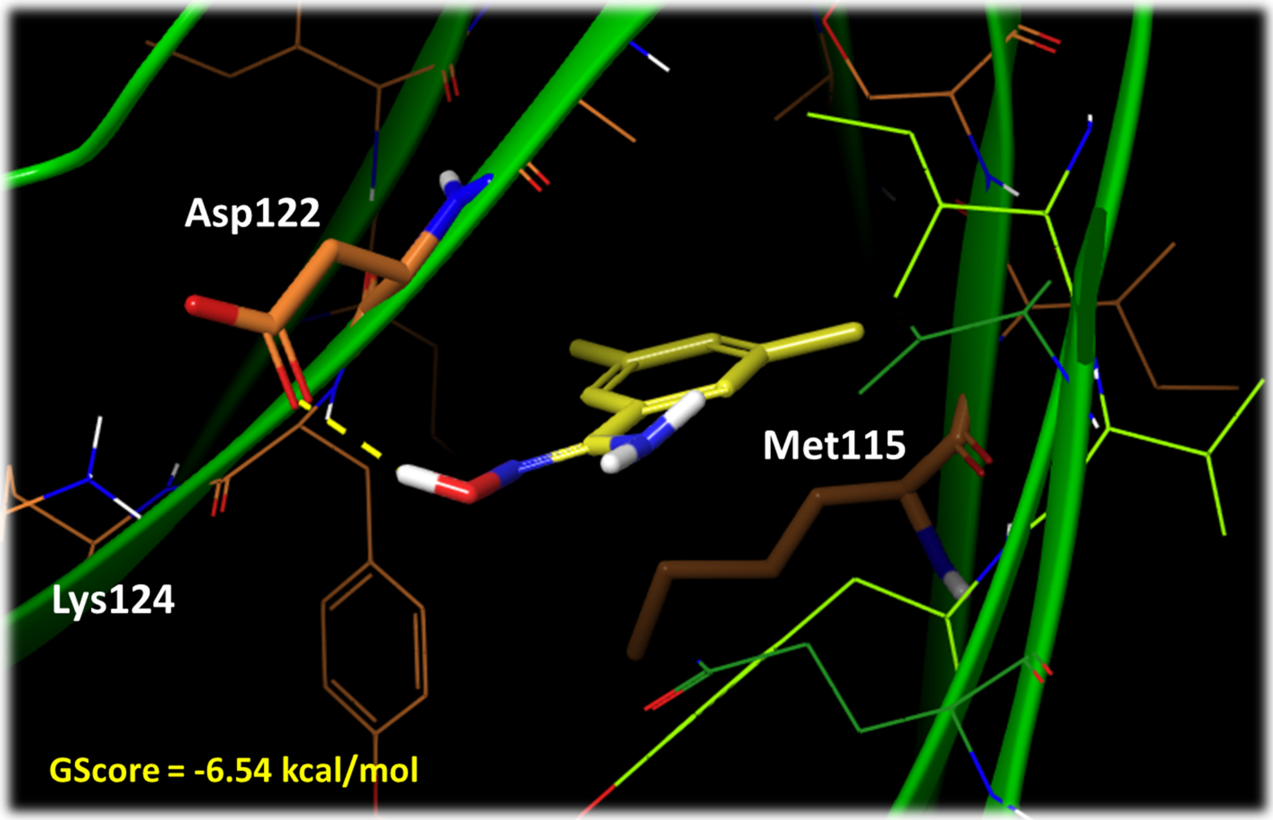

Figure S8. Binding mode of VIS1204 (**20**; yellow carbon atoms) to PD-L1. Residues of PD-L1 chain C are shown with orange carbon atoms (Asp122, Lys124, Tyr123). Residues of PD-L1 chain D are shown with green carbon atoms (Tyr56). Hydrogen bonds are shown as yellow dashed lines. Cation-π interactions are shown as green dashed lines.

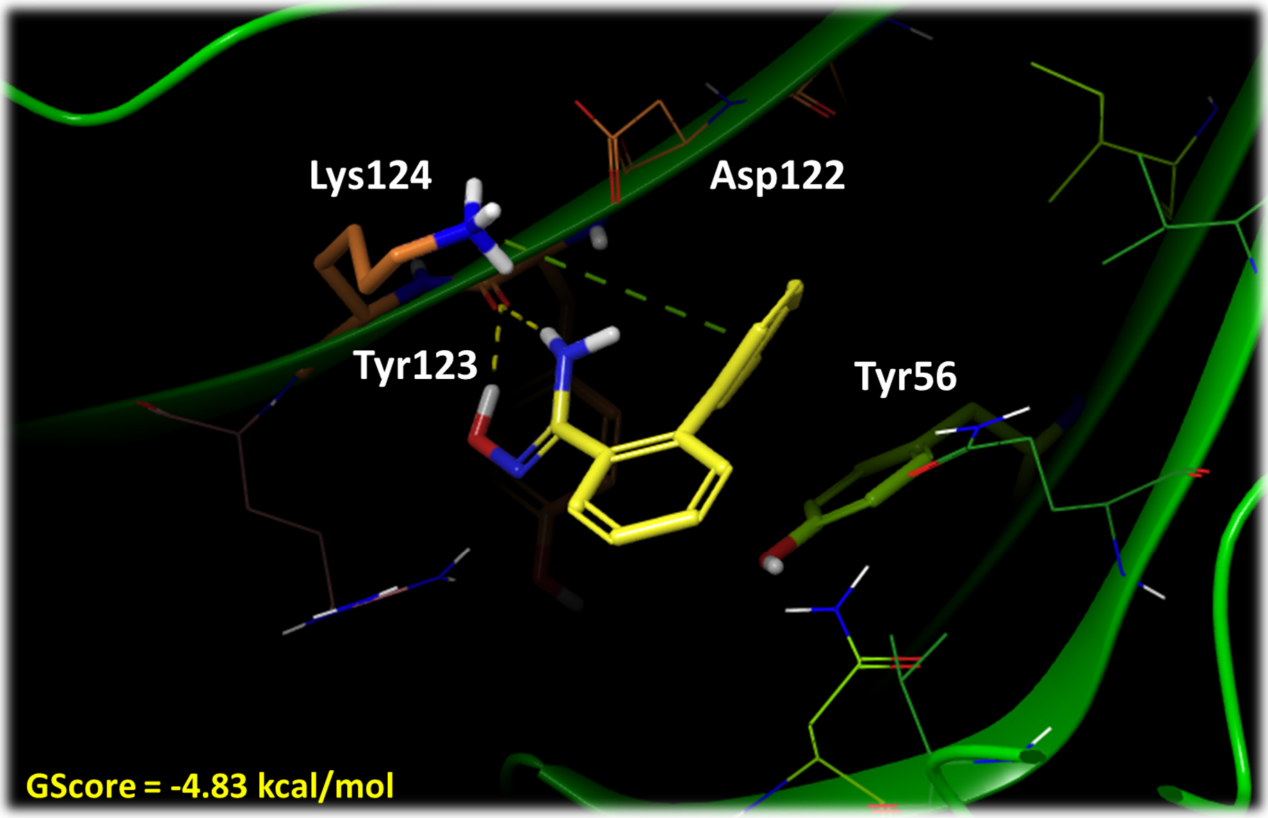

Table S1. Virtual hit compounds with chemical structure (smile format), molecular weight (MW, Dalton) and binding energy scores (Score, kcal/mol). Compounds VIS1075-VIS1084 belong to the subset of highly soluble fragments. Remaining compounds are from the internal subset of readily available small molecules. Red typed entries indicate putative hit compounds according to single-point binding assays.

Compounds (VIS)	SMILES	MW (Dalton)	Gscore (Kcal/mol)
VIS209	Cl.NCC(O)COCC1COC2=C(O1)C=CC=C2	239	-8.28
VIS228	NC[C@@H](O)c1cc(Cl)cc(Cl)c1	206	-8.05
VIS260	NC(=S)NNC(=O)c1cc(Br)ccc1	274	-6.99
VIS262	C[C@@H](O)c(n1)[nH]c(c12)cccc2	162	-7.48
VIS264	CC[C@@H](O)c([nH]1)[nH+]c(c12)cccc2	176	-7.63
VIS267	[NH3+]CC[C@@H](O)c1ccccc1	151	-7.55
VIS268	FC(F)(F)c1ccc(cc1)[C@H](O)C[NH3+]	205	-8.71
VIS270	[NH3+]C[C@@H](O)c1ccc(C)cc1	151	-7.24
VIS284	[NH3+]CCSc1c[nH]c(c12)cccc2	192	-7.94
VIS285	NCc1n[nH]c(=O)c(c12)cccc2	175	-7.05
VIS287	c1c[nH]c(=O)c(c12)cccc2O	161	-6.80
VIS300	OCC[NH2+]C[C@@H](O)Cn(c1cccc2)c(c3c12)cccc3	284	-7.02
VIS303	[H]/N=C(\N1)S[C@H](C1=O)CC(=O)Nc2cccc(c23)cccc3	299	-9.13
VIS307	[nH+]1ccn(C)c1[C@@H](O)c2cc(OC)c(cc2)OC	248	-8.26
VIS308	[NH3+]Cc(n1)n(C(C)C)c(c12)cccc2	189	-8.14
VIS309	NCc1cc(ccc1)-c2nccs2	190	-7.95
VIS310	ONC(=[NH2+])c1cc(C)c(O)c(C)c1	180	-7.22
VIS315	[NH3+]C[C@](C1)(O)CCc(c12)cccc2	177	-7.80
VIS328	C1COCC[NH+]1C[C@@H](O)COc2cccc(c23)cccc3	287	-8.42
VIS343	CNC[C@@H](O)Cn(c(C)c1C)c(c12)cccc2	232	-6.03
VIS344	CCN(CC)C[C@H](O)Cn(c(c1c23)C(=O)CCC1)c2cccc3	314	-6.79
VIS345	CCN(CC)C[C@H](O)Cn(c(C)c1C)c(c12)cccc2	274	-5.97
VIS346	c1cccc(c12)n(c(C)c2C)C[C@@H](O)CNCc3ccco3	298	-6.84
VIS471	c1cccc(c12)[nH]cc2CCNC(=O)Cc3csc(n3)NC(=O)c4c(=O)[nH]ccc4	421	-8.46
VIS542	COC1=CC2=C(C=C1OC)C(NCC2)C1=CC=C(O)C=C1	285	-6.21
VIS545	FC1=CC=C(CNC(=O)C2=CC=C3N=CNC3=C2)C=C1	269	-9.09
VIS549	CN1C(O)=NC2=C1C(=O)N(CC1=CC=CC=C1)C(=O)N2C	286	-7.80
VIS566	CC1=C(C(N2N=CN=C2N1)C1=CC=CN=C1)C(N)=O	256	-5.61
VIS568	NC(=O)CC1NC2=CC=CC=C2NC1=O	205	-7.54
VIS573	CC1=NC2=C(C=CC=C2)N1CC(N)=O	189	-7.54
VIS582	NCC(O)C1=CC2=C(OCC2)C=C1	179	-8.66
VIS584	CC1=CC=C(CSC2=NC=C(CO)N2CC(N)=O)C=C1	291	-9.22
VIS592	NC(=O)CN1C(CC2=CC=CC=C2Cl)=NC2=CC=CC=C12	300	-8.56
VIS597	FC1=CC=C(C=C1)C1=C(NN=N1)C(=O)NCC1=CC=CC=N1	297	-8.37
VIS599	NC(=O)C(=O)NCCC(O)C1=CC(Cl)=CC=C1	257	-8.15
VIS724	Cl.NC(CO)C(O)c1ccc(Cl)cc1	238	-7.55
VIS726	Oc1cc2cccc(O)c(=O)c2c(O)c1O	220	-5.86
VIS826	CN1C2=C(N(CC3=CC=C(C)C=C3)C(S)=N2)C(=O)N(C)C1=O	316	-6.05
VIS868	O=C(OC)C1=CC=C(O)C2=C1C3=C(OC2)C=CC=C3	256	-4.66
VIS871	CC(C1=C2C(C(OC)=O)=CC=C1)(C)OC3=C2C=CC=C3	268	-4.39
VIS872	CC(C1=C2C(C(OC)=O)=CC=C1O)(C)OC3=C2C=CC=C3	284	-4.36
VIS873	O=C(OC)C1=CC=C(O)C2=C1C3=C(OC2C)C=CC=C3	270	-4.73

VIS874	OC(C1=CC=C(O)C2=C1C3=C(OC2)C=CC=C3)=O	242	-4.82
VIS875	CC(C1=C2C(C(OC)=O)=CC(CCC)=C1)(C)OC3=C2C=CC=C3	310	-4.23
VIS876	O=C(OC)C1=CC=CC2=C1C3=C(OC2C4=CC=CC=C4)C=CC=C3	316	-5.87
VIS877	OC(C1=CC=CC2=C1C3=C(OC2)C=CC=C3)=O	226	-4.76
VIS878	O=C(OC)C1=CC=C(OC(C)=O)C2=C1C3=C(OC2)C=CC=C3	298	-5.08
VIS879	O=C(OC)C1=CC=C(C)C2=C1C3=C(OC2)C=CC=C3	254	-4.56
VIS32926	Nc1[nH]ncc1-c1ccc(F)cc1F	195	-7.71
VIS1058	O=C(NCC1=CC(CN2C=NC3=CC=CC=C23)=CC=C1)C1=CC=C(C=C1)C#N	366	-9.40
VIS1075	OC1CCC1NCc1ccncc1	178	-6.49
VIS1076	OC1CCCC1n1ccnc1	152	-7.00
VIS1077	CC(C)(C)C(=O)N1C[C@@H](O)[C@H](N2CCCC2)C1	240	-7.88
VIS1078	Cl.O=S1(=O)CCC2(CCCN2)CC1	189	-6.29
VIS1079	C[C@]1(O)CC[C@@H]1N1CC2CCCCC2C1	209	-7.61
VIS1080	CC1CCC2(CC1)NC(=O)NC2=O	182	-7.36
VIS1081	O=C1C(N2CCC(O)C2)CCN1CC(F)(F)F	252	-8.28
VIS1082	CC(C)(O)c1ncc[nH]1	126	-5.62
VIS1083	CN(C)C(=O)C1(F)CCNC1	160	-6.66
VIS1084	CN(C)C1CCC(O)CC1	143	-5.80

Table S2. Inflection temperature (T_i), initial ratio and differential (Δ) ratio of wt-PD-L1 and labelled NT650/PD-L1 at pH 7.2 and 6.2.

Sample	Ti [°C]	Initial Ratio	Δ Ratio
wt-PD-L1	61.36±0.15	0.67±0.01	0.29±0.02
NT650/PD-L1 pH 7.2	60.63±0.58	0.71±0.01	0.24±0.03
NT650/PD-L1 pH 6.2	60.73±0.49	0.66±0.01	0.26±0.03

Table S3. Calculated pKa ($_{calc}$pKa), experimental pKa and R^2 values of the potentiometric assays according to the Yasuda-Shedlovsky method.

**Compound**	**$_{calc}$pKa** (Marvin, v20.11; 2021)	**pKa**	**R^2**	**% MeOH**	**Temperature**
VIS310 (**9**)	5.60 ($_{calc}$pKa1)<br>9.18 ($_{calc}$pKa2)<br>11.02 ($_{calc}$pKa3)	5.65 ± 0.01<br>9.32 ± 0.02<br>n.d.	0.9981 (pKa1)<br>0.9971 (pKa2)<br>n.d.	50; 40; 30	25°C
VIS1200 (**16**)	5.24 ($_{calc}$pKa1)<br>9.33 ($_{calc}$pKa2)	5.26 ± 0.01<br>n.d.	0.9999 (pKa1)<br>n.d	45; 40; 35	25°C
VIS1201 (**17**)	5.38 ($_{calc}$pKa1)<br>8.70 ($_{calc}$pKa2)<br>10.52 ($_{calc}$pKa3)	5.35 ± 0.12<br>8.64 ± 0.01<br>n.d.	0.9999 (pKa1)<br>0.9999 (pKa2)<br>n.d	45; 40; 35	25°C
VIS1202 (**18**)	5.49 ($_{calc}$pKa1)<br>8.96 ($_{calc}$pKa2)<br>10.76 ($_{calc}$pKa3)	5.38 ± 0.01<br>8.97 ± 0.03<br>n.d.	0.9976 (pKa1)<br>0.9959 (pKa2)<br>n.d	45; 40; 35	25°C
VIS1203 (**19**)	5.59 ($_{calc}$pKa1)<br>8.94 ($_{calc}$pKa2)<br>10.67 ($_{calc}$pKa3)	5.38 ± 0.01<br>9.17 ± 0.02<br>n.d.	0.9998 (pKa1)<br>0.9971 (pKa2)<br>n.d.	45; 40; 35	25°C
VIS1204 (**20**)	4.99 ($_{calc}$pKa1)<br>9.02 ($_{calc}$pKa2)	5.30 ± 0.01<br>n.d.	0.9998 (pKa1)<br>n.d.	45; 40; 35	25°C

n.d.: not determined (pKa out of the range of values of SiriusT3, pKa > 12).
